# Correction: pUL21 is a viral phosphatase adaptor that promotes herpes simplex virus replication and spread

**DOI:** 10.1371/journal.ppat.1010225

**Published:** 2022-01-06

**Authors:** Tomasz H. Benedyk, Julia Muenzner, Viv Connor, Yue Han, Katherine Brown, Kaveesha J. Wijesinghe, Yunhui Zhuang, Susanna Colaco, Guido A. Stoll, Owen S. Tutt, Stanislava Svobodova, Dmitri I. Svergun, Neil A. Bryant, Janet E. Deane, Andrew E. Firth, Cy M. Jeffries, Colin M. Crump, Stephen C. Graham

The axis labels for Fig 3F are incorrect: the horizontal axis should have the range 0->100 nM. Please see the correct Fig 3 here.

**Fig 3:**
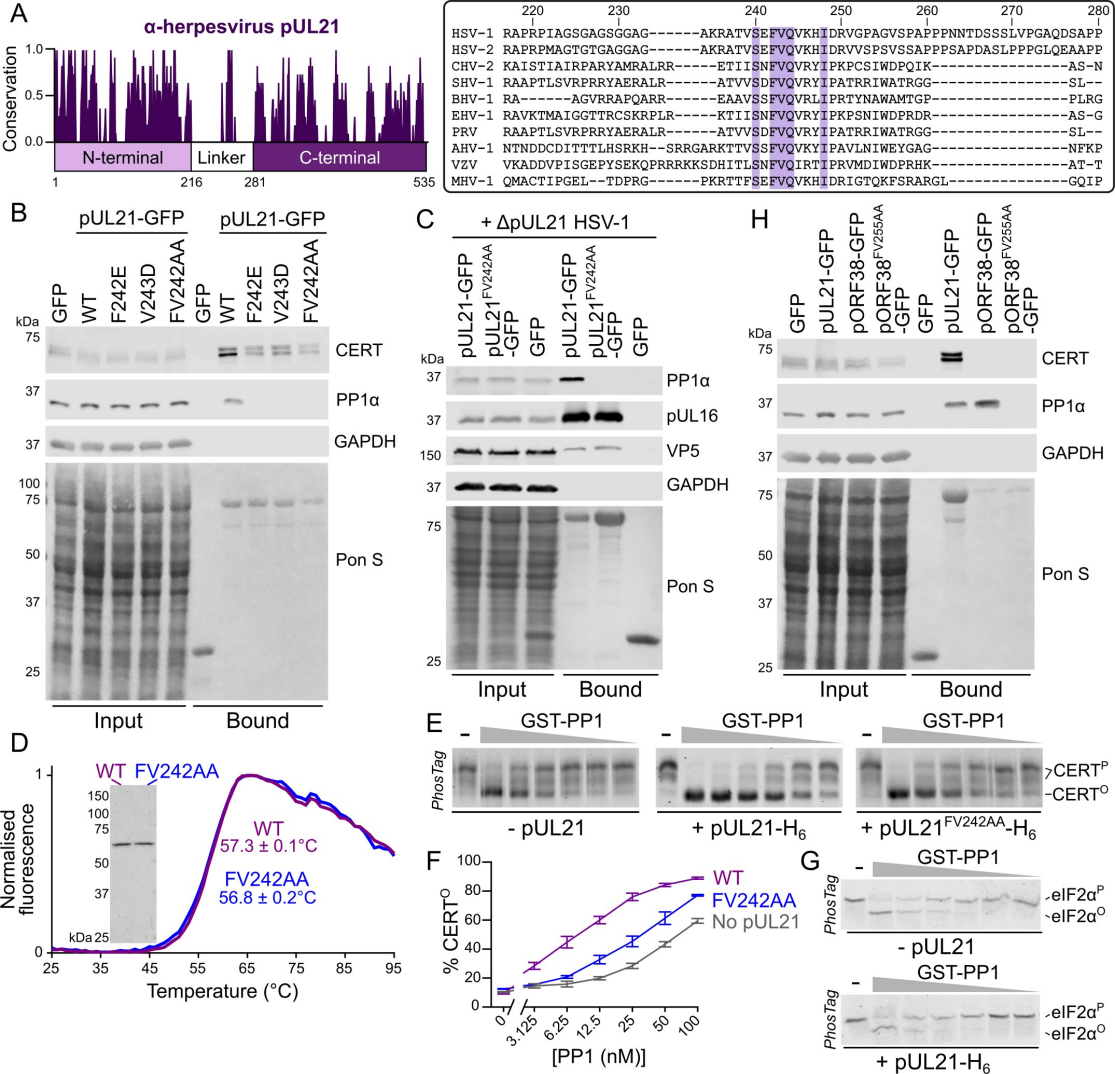
pUL21 recruits PP1 via a conserved motif in the linker region to accelerate CERT dephosphorylation. (**A**). Conservation of pUL21 across *Alphaverpesvirinae*. The following sequences were aligned using ClustalW and conservation calculated using Jalview (Abbreviation and Uniprot ID are shown in parentheses): HSV-1 (HSV1, P10205), HSV-2 (HSV2, G9I242), cercopithecine herpesvirus 2 (CHV2, Q5Y0T2), saimiriine herpesvirus 1 (SHV1, E2IUE9), bovine alphaherpesvirus 1 (BHV1, Q65563), equine herpesvirus 1 (EHV1, P28972), pseudorabies virus (PRV, Q04532), anatid herpesvirus 1 (AHV1, A4GRJ2), varicella-zoster virus (VZV, Q6QCT9), turkey herpesvirus (MHV1, Q9DPR5). Alignment across the linker region (residues 217–280 of HSV-1 pUL21) is shown with conserved residues highlighted. (**B**) HEK293T cells were transfected with plasmids expressing GFP, wild-type (WT) pUL21-GFP or pUL21-GFP with amino acid substitutions in the conserved motif. At 24 hours post-transfection the cells were lysed, subjected to immunoprecipitation using a GFP affinity resin, and captured proteins were subjected to SDS-PAGE and immunoblotting using the listed antibodies. Ponceau S (Pon S) staining of the nitrocellulose membrane before blocking is shown, confirming efficient capture of GFP-tagged proteins. (**C**) Plasmids expressing wild-type or mutant pUL21-GFP, or GFP alone, were transfected into HEK293T cells. At 24 hours post-transfection cells were infected with ΔpUL21 HSV-1 (MOI = 5). Cells were lysed 16 hours post-infection and subjected to immunoprecipitation, SDS-PAGE and immunoblotting as in (**B**). (**D**) Differential scanning fluorimetry of WT (purple) and FV242AA substituted (blue) pUL21-H_6_. Representative curves are shown. Melting temperatures (*T*_m_) is mean ± standard deviation (n = 3). Inset shows Coomassie-stained SDS-PAGE of the purified protein samples. (**E**) *In vitro* dephosphorylation assays using all-purified reagents. 0.5 μM CERT was incubated with varying concentrations of GST-PP1 (two-fold serial dilution from 100–3.1 nM) in the absence or presence of 2 μM pUL21-H_6_ (WT or FV242AA) for 30 min at 30°C. Proteins were resolved using SDS-PAGE where PhosTag reagent was added to enhance separation of CERT that is hyper- (CERT^P^) or hypo-phosphorylated (CERT^O^) and gels were stained with Coomassie. Images are representative of three independent experiments. (**F**) Quantitation of pUL21-mediated stimulation of CERT dephosphorylation, as determined by densitometry. Ratio of CERT^O^ to total CERT (CERT^O^ + CERT^P^) for three independent experiments is shown (mean ± SEM). (**G**) 0.5 μM phosphorylated eIF2α (eIF2α^P^) was subjected to *in vitro* dephosphorylation using varying concentrations of GST-PP1 (two-fold serial dilution from 200–6.3 nM) in the absence or presence of 2 μM pUL21-H_6_ as in (**E**). pUL21 does not enhance PP1-mediated dephosphorylation of eIF2α. (**H**) HEK293T cells were transfected with GFP, pUL21-GFP, the VZV homologue of pUL21 with a C-terminal GFP tag (pORF38-GFP), or with pORF38-GFP where amino acid in the conserved motif had been substituted with alanine. Cells were lysed at 24 hours post-transfection and subjected to IP, SDS-PAGE and immunoblotting as in (**B**).
