# Supplementary figures and images for: pUL21 is a viral phosphatase adaptor that promotes herpes simplex virus replication and spread
Source: PLoS Pathog. 2021 Aug 16;17(8):e1009824. doi: 10.1371/journal.ppat.1009824 (PMC8389370; doi:10.1371/journal.ppat.1009824)

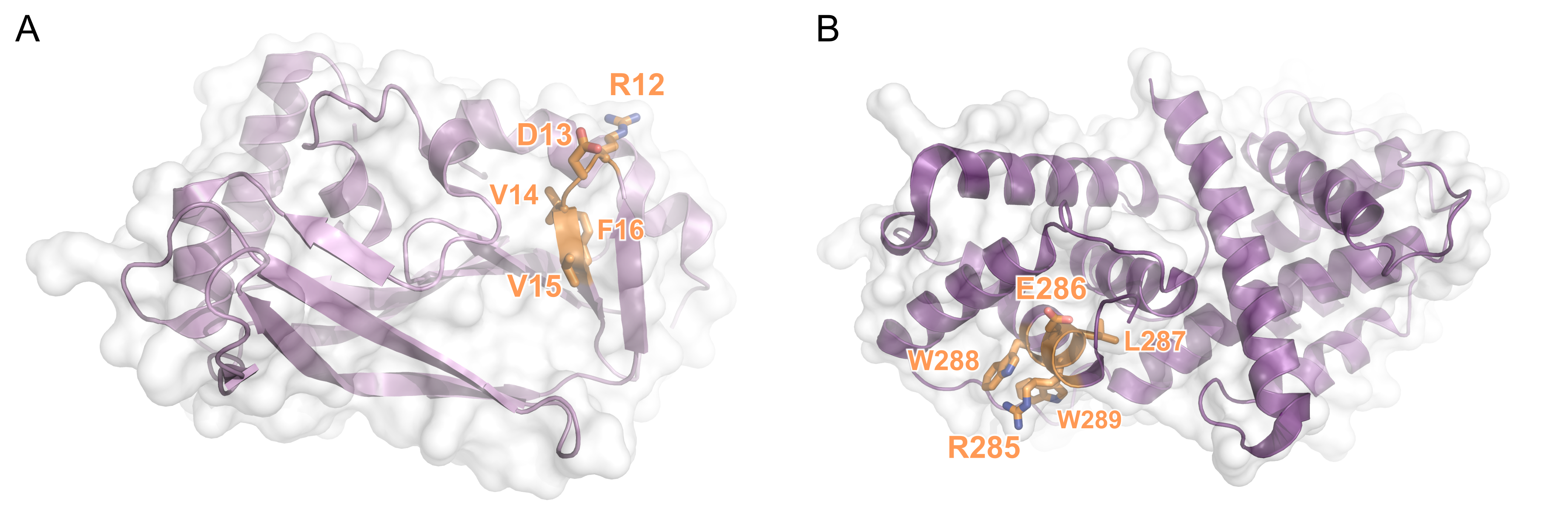

Supplement: S1 Fig — (A) N-terminal and (B) C-terminal domains of pUL21 are shown in cartoon representation with a semi-transparent molecular surface. Residues that match the consensus RVxF motif are shown as sticks with orange carbon atoms. For both domains, key hydrophobic residues in the potential RVxF motif are buried in the hydrophobic core of the protein and would be unable to interact with the RVxF-binding pocket of PP1 without very significant structural rearrangement. (TIF) [file ppat.1009824.s001.tif]

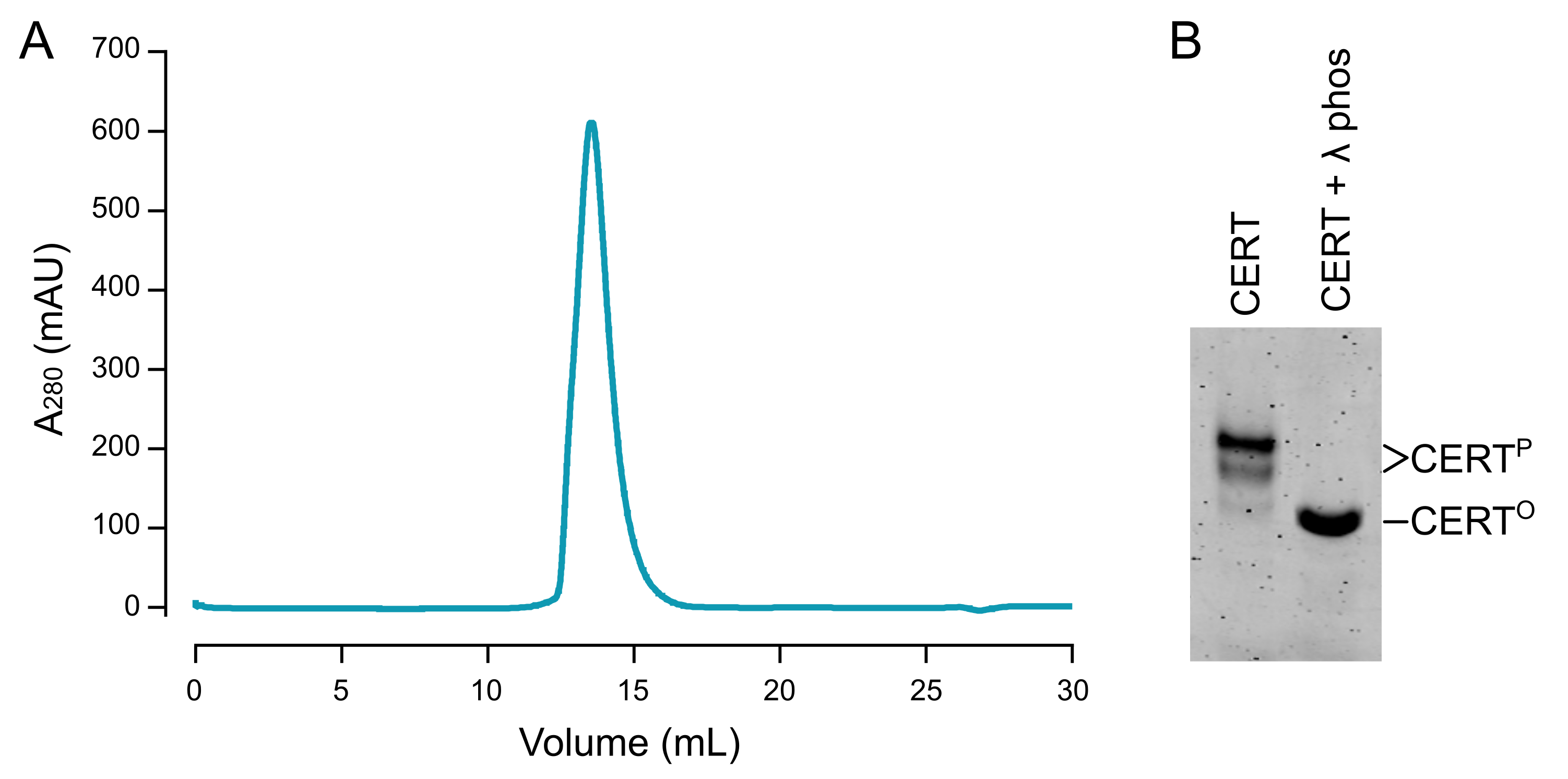

Supplement: S2 Fig — (A) SEC elution profile of Strep-II tagged CERT purified from Freestyle 293F cells grown in suspension culture. Following affinity purification using StrepTrap resin the protein was injected onto a Superose 6 10/300 column (GE Healthcare), from which it elutes as a single peak. (B) Purified CERT sample was dephosphorylated by incubation with lambda phosphatase (λ phos) in the presence of Mn2+ before being subjected to PhosTag SDS-PAGE analysis (25 μM PhosTag) and Coomassie staining, the PhosTag reagent acting to retard the electrophoretic mobility of phosphoproteins. The lambda phosphatase treated CERT (CERTO) migrates as a single band with significantly higher electrophoretic mobility than the untreated sample, confirming that CERT purified from suspension mammalian cells is hyperphosphorylated (CERTP). (TIF) [file ppat.1009824.s002.tif]

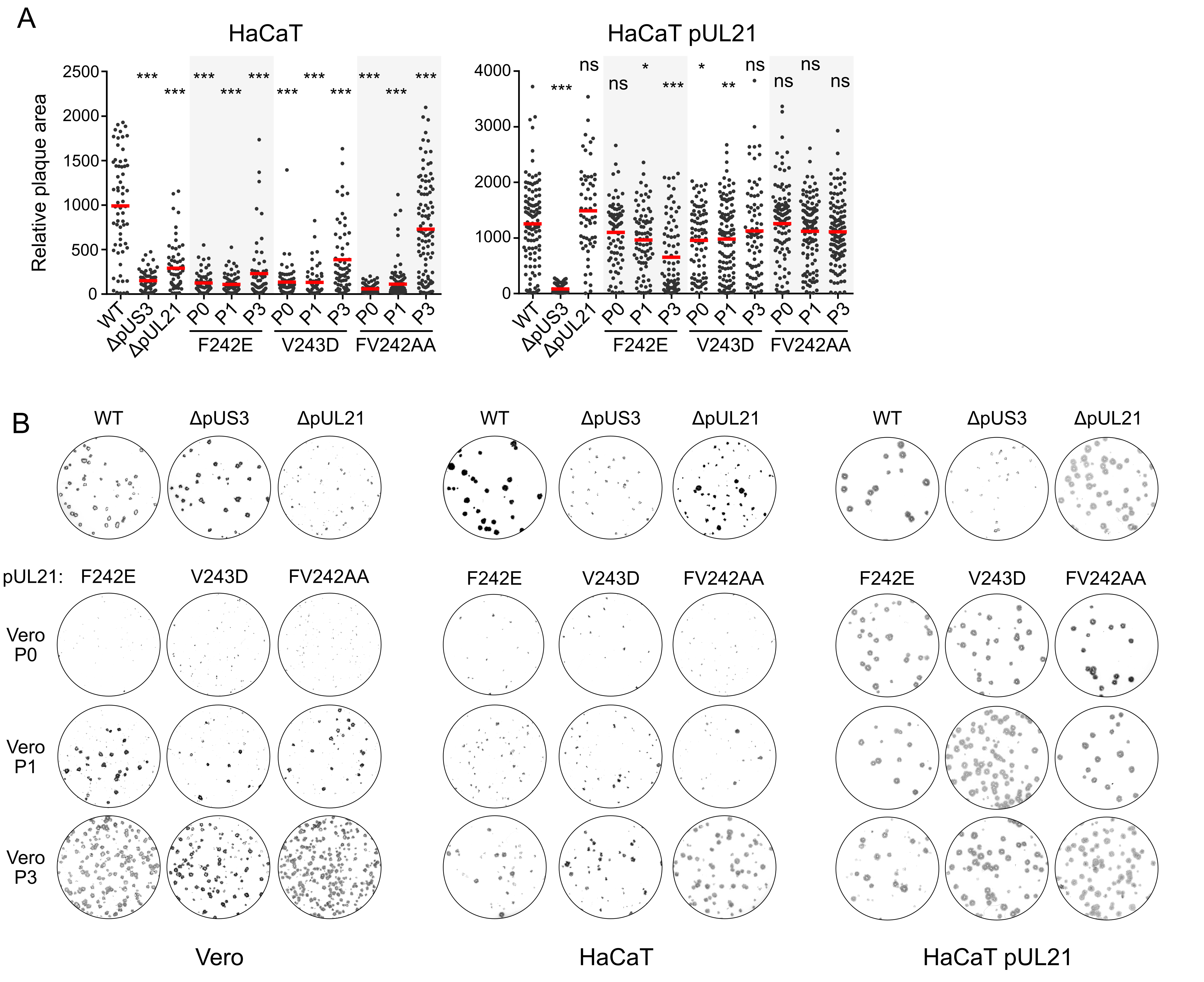

Supplement: S3 Fig — (A) Monolayers of HaCaT or HaCaT pUL21 cells were infected with 100 pfu of indicated viruses. Infected cells were overlaid with medium containing 0.6% carboxymethyl cellulose and incubated for 48 h before fixing and immunostaining with chromogenic detection. Relative plaque areas (pixels) were measured using Fiji. Mean plaque sizes (red bars) were compared to WT using one-way ANOVA with Dunnett’s multiple comparisons test (n = 59–119; ns, non-significant; *, P > 0.05; **, P < 0.01; ***, P < 0.001). (B) Plaque images used for quantitation in (A) and Fig 4A. (TIF) [file ppat.1009824.s003.tif]

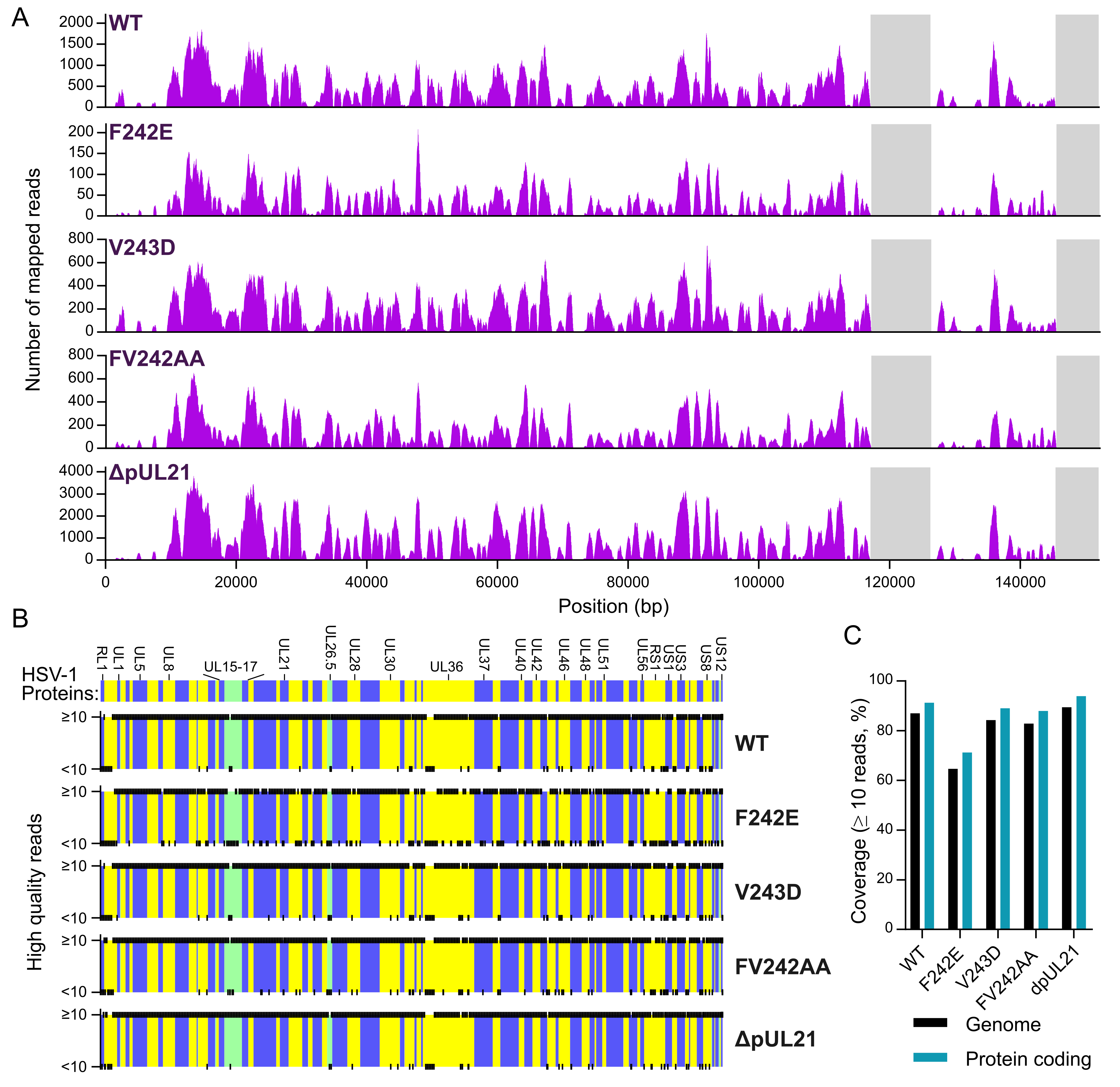

Supplement: S4 Fig — (A) Number of mapped reads across the HSV-1 genome is shown, with repeat regions excluded from the mapping highlighted in grey. (B) Regions of the HSV-1 protein coding regions where sufficient read depth (≥ 10 high quality reads) was obtained to analyse sequence variants. Black ticks denote nucleotide positions and alternating background colouring corresponds to HSV-1 genes, green denoting overlapping reading frames. (C) Percentage coverage of the HSV-1 genome (black) and coding regions (aqua) with ≥ 10 high quality reads. (TIF) [file ppat.1009824.s004.tif]

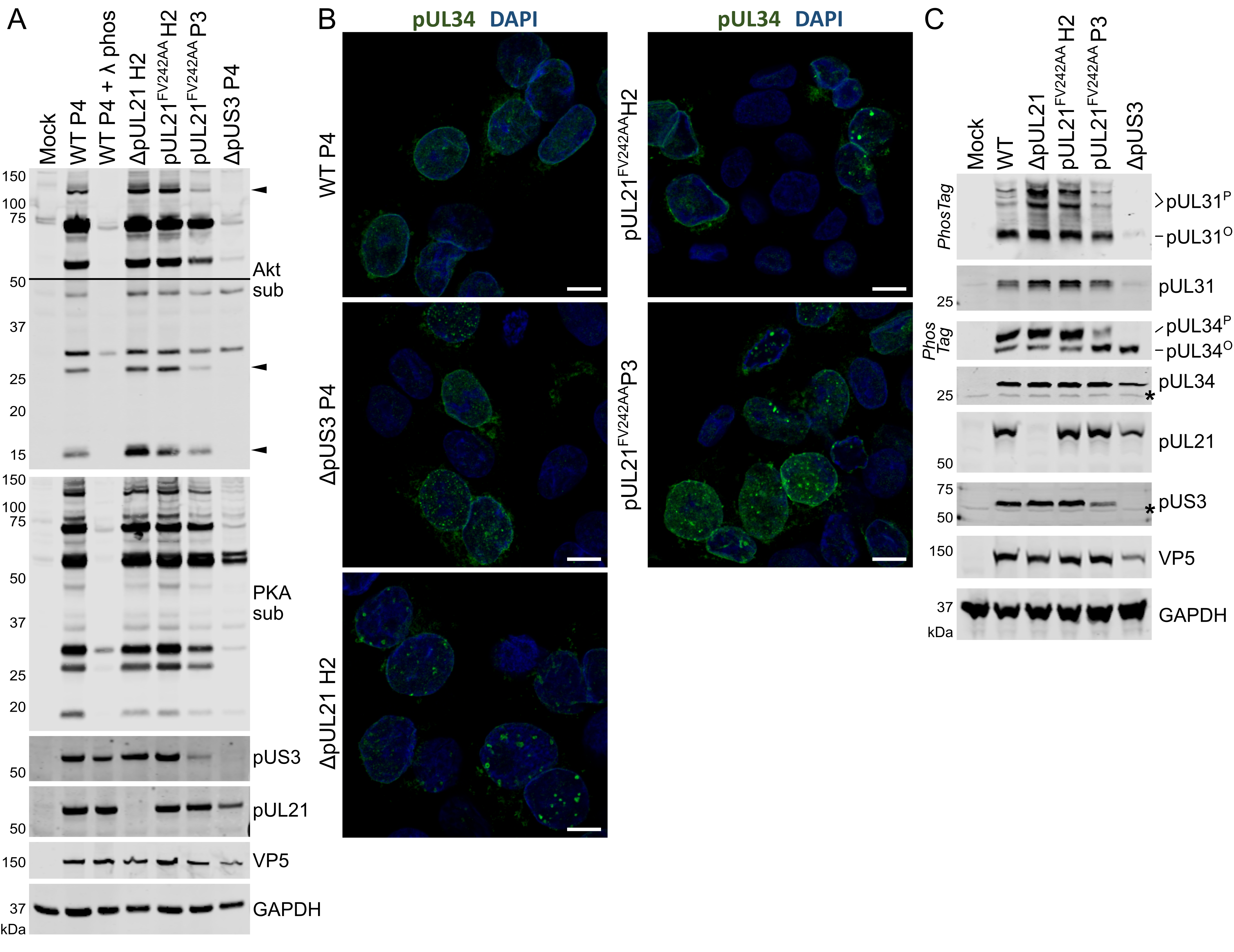

Supplement: S5 Fig — (A) HaCaT cells were infected and analysed as in Fig 6A. For the Akt sub immunoblot two exposures are shown, separated by a line, and phosphorylated Akt substrates more abundant in cells infected with virus lacking pUL21 that can recruit PP1 (ΔpUL21 H2 and pUL21FV242AAH2) are marked with arrowheads. (B). HaCaT cells were infected at MOI = 1 with WT or mutant HSV-1, prepared as in Fig 6A. Cells were fixed at 10 hpi and stained using an antibody that recognise pUL34 (green) plus DAPI (blue). The scale bar represents 10 μm. (C) HaCaT cells were infected at MOI = 5 with WT or mutant HSV-1 as listed. Lysates were harvested at 16 hpi and subjected to SDS-PAGE plus immunoblotting using the antibodies listed. The upper strips of the pUL31 and pUL34 blots depict SDS-PAGE where PhosTag reagent was added to enhance separation of hyperphosphorylated (pUL31P and pUL34P) and hypophosphorylated (pUL31O and pUL34O) forms of the proteins. Non-specific bands are indicated with an asterisk (*). (TIF) [file ppat.1009824.s005.tif]
