# Supplementary material for: pUL21 is a viral phosphatase adaptor that promotes herpes simplex virus replication and spread
Source: PLoS Pathog. 2021 Aug 16;17(8):e1009824. doi: 10.1371/journal.ppat.1009824 (PMC8389370; doi:10.1371/journal.ppat.1009824)
Supplement: S1 Table — Program version numbers are shown in parentheses. (DOCX) [file ppat.1009824.s006.docx]

**S1 Table. SAXS data collection and analysis parameters**. Program version numbers are shown in parentheses.

| *Data-collection parameters* |  |
| --- | --- |
| Radiation Source | Petra III (DESY, Hamburg, Germany) |
| Beamline | EMBL P12 |
| Detector | DECTRIS Pilatus 6M |
| X-ray wavelength (nm) | 0.124 |
| Sample-to-detector distance (m) | 3.0 |
| Temperature (°C) | 20 |
| Exposure time (s), Data frames (#) | 0.1, 47 |
| Measured protein concentrations (mg/mL) | 1.11–1.66 mg/ml |
| Measured *s*-range (nm^-1^) | 0.023–7.299 |
| Shannon channels (#), *s*_max_ (nm^-1^) | 39, 6.9 |
| Final working *s*-range (nm^-1^) | 0.067–2.94 |
| *Structural parameters* |  |
| *I*(0) (a.u.*) [from *p*(*r*)] | 0.028 ± 8.2×10^-5^ |
| Real-space *R*_g_ (nm) [from *p*(*r*)] | 4.6 |
| *I*(0) (a.u.*) (from Guinier) | 0.028 ± 7.3×10^-5^ |
| *R*_g_ (nm) (from Guinier) | 4.2 |
| *D*_max_ (nm) | 18.1 |
| Porod volume estimate (Vp, nm^3^) | 90 |
| *Molecular-mass (*M_r_*) determination* |  |
| *M_r_* from Bayesian consensus (kDa) | 60 |
| *M_r_* credibility interval (kDa) | 58–66 |
| Expected *M*_r_ from sequence (kDa) | 58.7 |
| *Software employed* |  |
| Primary data reduction | *SASFLOW* |
| Data processing | *PrimusQT/GNOM(5.0)* |
| Ensemble modelling | *EOM(2.1)* |
| *Small Angle Scattering Biological Data Bank* |  |
| SASBDB accession codes | SASDKW8 |
