# Supplementary material for: pUL21 is a viral phosphatase adaptor that promotes herpes simplex virus replication and spread
Source: PLoS Pathog. 2021 Aug 16;17(8):e1009824. doi: 10.1371/journal.ppat.1009824 (PMC8389370; doi:10.1371/journal.ppat.1009824)
Supplement: S2 Table — Regions of the primer homologous to pUL21 are shown in italic, with homologous regions that will recombine being underlined. Mutations are in bold and the region homologous to pEPkan-S is in lower case. (DOCX) [file ppat.1009824.s007.docx]

**S2 Table. Primers used to make mutant HSV-1 strains by two-step Red recombination.** Regions of the primer homologous to pUL21 are shown in *italic*, with homologous regions that will recombine being underlined. Mutations are in **bold** and the region homologous to pEPkan-S is in lower case.

| **Mutant** | **Forward primer (5′ to 3′)** | **Reverse primer (5′ to 3′)** |
| --- | --- | --- |
| ΔpUL21 | *GCACTACCGGGACGTTGTGTTTTACGTCACAACGGACCGA***TGATAGTAA**G*CTTTGTGTGCGGGGGGTGTG*aggatgacgacgataagtaggg | *CGGCCGCCCCACGGAATAAACACACCCCCCGCACACAAAG*C**TTACTATCA***TCGGTCCGTTGTGACGTAAA*caaccaattaaccaattctgattag |
| pUL21^F242E^ | *GTCGGGGGGCGCGGGCGCCAAGCGGGCCACCGTCAGCGAG***GAG***GTGCAAGTCAAACACATTGA*aggatgacgacgataagtaggg | *CGCCAGCGGGGCCCACGCGGTCaatGTGTTTGACTTGcac***CTC***CTCGCTGACGGTGGCCCGCT*caaccaattaaccaattctgattag |
| pUL21^V243D^ | *GGGGGGCGCGGGCGCCAAGCGGGCCACCGTCAGCGAGTTC***GAC***CAAGTCAAACACATTGACCG*aggatgacgacgataagtaggg | *AAACGCCAGCGGGGCCCACGCGGTCaatGTGTTTGACTTG***GTC***gaaCTCGCTGACGGTGGCCC*caaccaattaaccaattctgattag |
| pUL21^FV242AA^ | *GTCGGGGGGCGCGGGCGCCAAGCGGGCCACCGTCAGCGAG****GCAGCA****CAAGTCAAACACATTGACCG*aggatgacgacgataagtaggg | *AAACGCCAGCGGGGCCCACGCGGTCaatGTGTTTGACTTG***TGCTGC***CTCGCTGACGGTGGCCCGCT*caaccaattaaccaattctgattag |
| ΔpUS3 | *CACCACACCACCCGGCGATGCCGAGCGCCTGTGTCATCTGTGATCTTCGAGACTGCCGTC*aggatgacgacgataagtaggg | *GAGAACAAGGACGCGTTGTGGACGGCAGTCTCGAAGATCACAGATGACACAGGCGCTCGG*caaccaattaaccaattctgattag |
