# Supplementary material for: pUL21 is a viral phosphatase adaptor that promotes herpes simplex virus replication and spread
Source: PLoS Pathog. 2021 Aug 16;17(8):e1009824. doi: 10.1371/journal.ppat.1009824 (PMC8389370; doi:10.1371/journal.ppat.1009824)
Supplement: Attachment — Submitted filename: 210717 Benedyk Reviewer Responses.docx [file ppat.1009824.s009.docx]

Reviewer #1: This excellent paper convincingly shows that pUL21 of herpes simplex virus is an adapter that bridges PP1 to ceramide transport protein (CERT), an observation that likely activates its activity. The structure of pUL21 is loosely defined and is compatible with such an adapter, and the binding activity on both pUL21 and CERT are mapped. The activation of CERT may have implications for virus budding or envelopment activity.

Separately, the study shows that pUL21 helps decrease US3-mediated phosphorylation of the nuclear egress complex (NEC) comprising pUL31 and pUL34. Interesting data show second site mutations in US3 that arise upon passage of the UL21 virus in noncomplementing cells that restore viral replication and plaque size.

The scope of work is remarkable with structural biology, virology, and biochemistry all underlying the highly significant and strongly supported conclusions. The abstract is pleasingly understated, and the discussion concise.

Although the CERT and NEC activities might be viewed as separate studies, the fact that there is more than one substrate that can bind pUL21 to be de-phosphorylated further shows pUL21’s role as an adapter with limited specificity of substrate.

I do not have any significant concerns that need to be addressed. This is an important piece of work that will likely have implications for, and develop an appreciation for, an underappreciated regulatory mechanism.

We thank the reviewer for their consideration of the manuscript and supportive comments.

Reviewer #2: In this work the authors report on the function of the UL21 protein of herpes simplex virus. Previous reports have already described its interaction with UL16, and separate possible roles via an interaction with UL11 and gE, among other possible functions. Here the authors describe and interaction, initially via transient transfection of the protein into 293 cells, between UL21 and the catalytic subunit of the cellular phosphatase, PP1. In the same analysis they also describe an interaction with the protein CERT, involved in ceramide-transport.

They then go on to identify specific regions and determinants within pUL21 for interaction required for PP1 direct binding. Following that they construct viral mutants that either lack pUL21 entirely or contain disruptions of the PP1 binding site and characterise plaque formation and replication parameters. Finally they demonstrate that while pUL21 mutants that lack the PP1 binding site initially grow extremely poorly, such mutants rapidly revert to a virtually normal phenotype. But intriguingly from the sequence analysis they show that the reversion is not due to reversion with pUL21 itself but rather due to mutation in the pUS3 kinase gene and disruption of its kinase activity. This provides very convincing evidence for a functional interaction between pUL21 and its PP1 binding activity on the one hand, and pUS3 kinase activity on the other. With convincing analysis of one of the main pUS3 targets, that is pUL34 and its role in nuclear egress, the authors provide very convincing evidence for a role of pUL21 in modulating and indeed tuning the phosphorylation of pUL34 to optimise nuclear egress activity.

Altogether this is an extremely convincing and interesting piece of work. The data are very sound, quantitatively rigorous and overall provides strong support both for the relevance of pUL20/PP1 interaction generally and more specifically for a role of this complex in moderating the pUS3 kinase activity on certain of its substrates, particulalry pUL34.

We thank the review for their positive appraisal of our work.

Minor points are as follows.

1. The authors report the direct binding of pUL21 and PP1 initially in transient transfection assays in 293 cells and also by interaction with purified proteins expressed in bacteria. But nowhere did I see evidence for the direct interaction by co-precipitation in virus-infected cells, and this would be useful.

In Figure 3C we show co-precipitation of PP1 in the context of infection. Cells ectopically expressing GFP-tagged pUL21 (WT or FV242AA) were super-infected with ΔpUL21 HSV-1. PP1 co-precipitates with WT pUL21, but not with pUL21^FV242AA^. Furthermore, we note that interactions observed using co-immunoprecipitation experiments in the context of infection can be indirect (mediated by other viral or cellular components). Our biochemical assays using all-purified components confirm that the interactions between pUL21, PP1 and CERT are genuinely direct. Figure 1C confirms that purified pUL21 binds PP1 and CERT directly, and Figure 3E confirms that this interaction promotes pUL21-mediated CERT dephosphorylation.

2. Associated with the point above would be the simple description of the total abundance of PP1, and likely all of its isoforms, during viral infection, for example in a time course by Western blotting. The same could be said of CERT.

We have interrogated our published HSV-1 whole cell proteomics analysis (Soh et al (2020) Cell Reports) and confirm that neither CERT (COL4A3BP) not any of the PP1 catalytic domain isoforms (PPP1CA, PPP1CB, PPP1CC) change significantly in abundance over the course of infection – please see graph below. We have added a sentence to this effect to the Results.

3. The initial finding was for a direct interaction with the catalytic subunit of PP1. It would be worth discussing whether the regulatory subunits were also co-precipitated, whether they think pUL21 interacts the holo- enzyme, or just the catalytic subunit, and since the catalytic subunit is present in very numerous combinations of holoenzymes, how would an interaction with the catalytic subunit be substrate specific.

We have re-assessed the pUL21 interactomics data (Figure 1A and Supplementary Data) and we do not observe significant co-precipitation of any cellular PP1 regulatory subunits with GST-pUL21. Furthermore, our *in vitro* dephosphorylation experiments using all purified components (Figure 3E) confirm that pUL21 promotes CERT dephosphorylation in the presence of just the PP1γ catalytic subunit (i.e. in the absence of cellular regulatory subunits). From these experiments we infer that pUL21 is itself a novel viral PP1 regulatory subunit. Given the novelty of the PP1-interacting motif utilised by pUL21 (the TROPPO motif) it remains unknown whether pUL21 would compete with cellular PP1 regulatory subunits (a.k.a. adaptor proteins, PIPs or RIPPOs) or could work synergistically with them. We have added a sentence highlighting this interesting future research question to the penultimate paragraph of the discussion.

4. For the enhancement of phosphatase activity on CERT, in figure 3E, where the authors state the range of PP1 concentrations, they don't date the stepwise increments which would be useful to try and assess quantitatively the fold enhancement by adding pUL21. It is noteworthy that this is in the absence of regulatory subunits and one wonders what the effect would be then, and how the 2uM pUL21 compares with eg addition of regulatory subunits

We apologise that the description of our experimental setup was unclear – we have updated the legend of Figure 3E to confirm that we performed a two-fold serial dilution of GST-PP1. We have also included a figure quantitating the dephoshorylation effect, with exact PP1 concentrations shown (see response to Reviewer 3, below).

With regard to the concentration of pUL21, 2 uM was chosen so as to give 4-fold molar excess compared to CERT, 0.5 uM CERT having been chosen as this was empirically determined to be the lowest concentration affording reliable detection of both CERT^P^ and CERT^O^ by Coomassie-stained SDS-PAGE. We concede that 2 uM pUL21 is a large excess when compared to published interactions between PP1 and regulatory subunits, with affinities in the low-to-mid nM range. However, 2 uM pUL21^FV242AA^ has dramatically reduced ability to stimulate CERT dephosphorylation (Figures 3E and 3F) and 2 uM wild-type pUL21 is unable to stimulate eIF2α dephosphorylation (Figure 3G). Taken together, these data confirm that pUL21 specifically enhances PP1-mediated CERT dephosphorylation rather than non-specifically enhancing PP1 activity by, for example, molecular crowding. Determining the structure and exact affinity of the PP1:pUL21 interaction is an interesting topic of future research.

Overall this is a very good piece of work not only for the detail on mechanistic insight into pUL21, but also for the principal of one viral protein, via recruitment of the cellular phosphatase, fine tuning the activity of another viral protein in this case kinase. This point is made all the more convincing by in vivo analysis of viral mutants and the convincing demonstration of the revertants in a different gene.

Reviewer #3: Benedyk et al., present interesting and compelling data showing that the HSV UL21 protein interacts with and acts as an adapter for protein phosphatase 1 (PP1). They identify a motif in pUL21 that mediates interaction with PP1 and show that mutation of this motif impairs virus growth and spread, and furthermore that minimal selection of virus that have mutations in this motif yields viruses that both replicate and spread better than the parental mutant. Selection is correlated with appearance of mutations in the US3 coding sequence, suggesting that loss of pUS3 kinase activity may compensate for loss of phosphatase activity on specific substrates, including proteins of the nuclear egress complex. This is a very interesting paper, and well worth publication in this journal. I have some criticisms with regard to specific pieces of data and interpretation as listed below.

We thank the reviewer for their enthusiasm for our work.

1. The authors analysis of variants that arise in response to pUL21 mutation is based on sequencing of the variant populations rather than individual, plaque-purified viruses. This has the advantage that they can sample a lot of the variation in the population without sequencing many viruses. The disadvantage is that they end up with no idea whether any of these variants is sufficient to suppress the UL21 mutant phenotype. For example, it is possible that the US3 mutations are each paired with something else that is necessary for the suppressive effect. The possibility of interactions between different mutations within these populations might have been addressed directly by generation of a recombinant double UL21/US3 mutant. While these data are sufficiently interesting without this, the authors should discuss the possibility of multiple mutation interactions and appropriately soften their conclusions about US3 involvement in genetic compensation.

We appreciate the important point raised by the reviewer, that additional mutations in the genome that are genetically linked to the pUS3 mutations may be at low-enough frequencies to make them ‘invisible’ in our analysis of mutant populations. We have added a note to the discussion describing this possibility and softened the assertion in our abstract accordingly.

2. There are two instances where the authors make semi-quantitative conclusions from western blots where quantitation of multiple experiments is not presented. The first of these is in lines 203-204, referring to Figure 3E. The authors state that “Addition of purified wild-type pUL21-H6 dramatically lowers the concentration of GST-PP1γ required for efficient CERTP dephosphorylation but addition of pUL21FV242AA-H6 does not …”. The differences pointed to are not dramatic and are, in fact, subtle enough that quantitation is required. The second instance is in Figure 6C, where the differences between pUL31 phosphorylation in WT and UL21 mutant conditions is also subtle enough to require quantitation to support the statement that the extent of hyperphosphorylation of pUL31 is increased in these conditions.

We appreciate the reviewer’s careful analysis of our experimental data. We have included an additional panel in Figure 3 (panel F) where we have quantitated the dephosphorylation of purified CERT^P^ in three independent experiments, confirming greater acceleration of CERT^P^ in the presence of WT pUL21 than in the presence of pUL21^FV242AA^.

With regards to Figure 6C, we have performed PhosTag analysis of pUL31 phosphorylation following infection on multiple independent occasions in both HaCaT and Vero cells and we consistently observe the same qualitative result (more pUL31^P^ following infection with ΔpUL21 HSV-1 or unadapted pUL21^FV242AA^ HSV-1 than with WT or adapted pUL21^FV242AA^ HSV-1). However, we do not observe discrete bands for the different pUL31 phosphovariants and the extent of protein migration varies between individual PhosTag gels, complicating comparative quantitation. To aid the reader in visualising the changes in pUL31 phosphovariant abundance shown in Figure 6C we have plotted a signal intensity profile for each lane (Figure 6D). This clearly illustrates increased prevalence of phosphorylated pUL31 in ΔpUL21 HSV-1 or unadapted pUL21^FV242AA^ HSV-1 infected Vero cells (denoted with arrowheads in Figure 6D).

3. In lines 326-27 the authors state that The subcellular localization of pUL21 was broadly similar between this mutant and the wild-type virus, confirming that the conserved motif is not required for recruitment of pUL21 to the nuclear rim…” Recruitment of pUL21 to the nuclear rim is not at all evident in the lower two rows of panel B. In fact, magenta staining is essentially invisible in these images. The authors need better images or rigorous quantitation of the nuclear envelope localization by co-localization with pUL34 or with lamins.

We apologise that the images in the original manuscript were insufficiently clear. We have adjusted the contrast for the anti-pUL21 (magenta) channel of the pUL21^FV242AA^ HSV-1 infected cells in Figure 6B to clearly demonstrate the nuclear rim staining of pUL21. As stated in the text, the staining for pUL21 in these mutants is broadly similar to the wild-type but not identical as there is an increase in the number of cytoplasmic pUL21 punctae observed.

Minor point:

In Figure 4E, the authors indicate the positions of residues S207 and D208 in pUS3. I think they mean to label them D207 and S208.

We thank the reviewer for spotting this typo. The figure has been updated accordingly.

Part II – Major Issues: Key Experiments Required for Acceptance

Please use this section to detail the key new experiments or modifications of existing experiments that should be absolutely required to validate study conclusions.

Generally, there should be no more than 3 such required experiments or major modifications for a "Major Revision" recommendation. If more than 3 experiments are necessary to validate the study conclusions, then you are encouraged to recommend "Reject".

Reviewer #1: none

Reviewer #2: This is an excellent piece of work. I see no major issues

Reviewer #3: Quantitation of western blot data in Figures 3E and 6C.

The requests of reviewer 3 have been addressed as described above.

________________________________________

Part III – Minor Issues: Editorial and Data Presentation Modifications

Please use this section for editorial suggestions as well as relatively minor modifications of existing data that would enhance clarity.

Reviewer #1: none

Reviewer #2: 1. Providing evidence for the direct interaction by co-precipitation in virus-infected cells, and this would be useful.

2. Associated with this would be the simple description of the total abundance of PP1, and likely all of its isoforms, during viral infection, for example in a time course by Western blotting. The same could be said of CERT.

3. The initial finding was for a direct interaction with the catalytic subunit of PP1. It would be worth discussing whether the regulatory subunits were also co-precipitated, whether they think pUL21 interacts the holo- enzyme, or just the catalytic subunit, and since the catalytic subunit is present in very numerous combinations of holoenzymes, how would an interaction with the catalytic subunit be substrate specific.

4. For the enhancement of phosphatase activity on CERT, in figure 3E, where the authors state the range of PP1 concentrations, they don't date the stepwise increments which would be useful to try and assess quantitatively the fold enhancement by adding pUL21. It is noteworthy that this is in the absence of regulatory subunits and one wonders what the effect would be then, and how the 2uM pUL21 compares with eg addition of regulatory subunits

Reviewer #3: (No Response)

These concerns of reviewer 2 have been addressed as described above.
